# Leadership or luck? Randomization inference for leader effects in politics, business, and sports

**DOI:** 10.1126/sciadv.abe3404

**Published:** 2021-01-20

**Authors:** Christopher R. Berry, Anthony Fowler

**Affiliations:** Harris School of Public Policy, University of Chicago, Chicago, IL, USA.

## Abstract

Anecdotal evidence suggests that some leaders are more effective than others but observed differences in outcomes between leaders could be attributable to chance variation. To solve this inferential problem, we develop a quantitative test of leader effects that provides more reliable inferences than previous strategies, and we implement the test in the settings of politics, business, and sports. We find significant effects of political leaders, particularly in nondemocracies. We find little evidence that chief executive officers influence the performance of their firms. In addition, we find clear evidence that sports coaches matter for a wide range of outcomes in football, basketball, baseball, and hockey.

## Teaser

A new method finds that leaders are highly consequential in politics and sports but not business.

## INTRODUCTION

The U.S. economy grew at an annual rate of over 6% during Harry Truman’s second term, faster than under any other postwar president. Under the leadership of Rahm Emanuel, homicides in Chicago increased 70% between 2014 and 2018. Bill Belichick led the New England Patriots to six Super Bowl titles in 18 seasons. After Jonathan Schwartz became chief executive officer (CEO) of Sun Microsystems, the firm’s share price fell from 27 to 4 dollars.

How much credit do leaders such as Truman and Belichick deserve for the outcomes that happened on their watch? How much blame should fall at the feet of those similar to Emanuel and Schwartz? Do the decisions and actions of leaders change the course of events, or are some merely (un)lucky, holding their position at a time when other factors would have generated largely the same outcomes regardless of who sat at the helm? This question, whether leaders matter, has fascinated scholars for centuries. Today, universities offer advanced degrees in leadership, and airport bookstores feature bestsellers on the topic, implying a settled conclusion that leaders matter. However, there is little rigorous empirical evidence on the effects of leaders.

Here, we introduce a new method for statistically testing leader effects, which has several advantages relative to other methods that have been used previously. We call this method randomization inference for leader effects or RIFLE. After assessing the advantages and limitations of our method, we demonstrate its substantive value by estimating effects of political leaders, CEOs, and sports coaches on various outcomes.

To preview our results, we find strong evidence that world leaders influence their economies, more so in autocracies than in democracies. We find that governors influence fiscal policy and crime, but not overall economic prosperities, and we find no evidence that mayors are similarly influential. We find little evidence that CEOs systematically affect the performance of the firms that they lead. Our estimated leader effects are largest in the realm of sports, at both the professional and collegiate levels, where we find evidence that coaches significantly influence their team’s records and various additional measures of on-field performance.

The subject of leadership has fascinated scholars at least back to the ancient Greeks. In addition, for only as long, scholars have debated whether leaders matter in shaping outcomes for better or worse. Thucydides chronicled the great leaders, such as Pericles, whose particular decisions and abilities, in his view, determined the outcome of the Peloponnesian War. Meanwhile, Plato extolled the virtues of the philosopher king while advocating a system of education and selection that would make any individual leader replaceable by another who would make similar decisions.

In the 19th century, Carlyle (*1*) advanced the still influential view that “the history of the world is but the biography of great men.” However, his contemporary, Marx (*2*), argued that historically determined social and economic forces constrain the choices available to leaders, making any individual relatively unimportant in the course of events.

More recently, the role of political leadership has been overshadowed in economics and political science by the role of institutions in determining the fate of nations. Building on the work of North (*3*), a dominant theme in the contemporary literature on economic development is that good institutions are a major contributor to long-run economic growth (*4*), although empirically identifying the effects of institutions is not unproblematic (*5*).

A seminal paper by Jones and Olken (*6*) rekindled interest in the empirical study of political leadership by providing the most credible evidence to date that political leaders matter. They use unexpected deaths of world leaders while in office as a source of exogenous variation in leadership. They show that the rate of economic growth in a country changes when a leader dies in office. Their key empirical test is whether the change in economic growth between the last 2 years of one leader’s tenure and the first 2 years under the successor is greater than would be expected by chance. They find strong evidence of abnormal variation in growth around exogenous leader transitions, which implies that leaders matter. They show further evidence that unexpected leader turnover leads to changes in economic growth in autocracies but not democracies.

The findings of Jones and Olken (*6*) have been extended in several ways by subsequent authors. Besley *et al.* (*7*) find that educated leaders particularly exert a positive effect on economic growth. Within the Jones-Olken framework, they show that a transition from a more educated to a less educated leader results in a reduction in economic growth, while a transition from a less educated to a more educated leader leads to a boost in economic growth. Yao and Zhang (*8*) analyze the effects of city leaders in China on local economic growth, taking advantage of the fact that leaders regularly move, so that the same person may be the mayor of multiple cities over the course of her career. Yao and Zhang (*8*) use all leader transitions in their analysis rather than identifying unexpected transitions as in Jones and Olken (*6*), and they find mixed results depending on the statistical test they consider. Easterly and Pennings (*9*) challenge Jones and Olken’s (*6*) conclusion that leaders matter more in autocracies than in democracies. Specifically, Easterly and Pennings (*9*) estimate leader fixed effects in a growth model and show that the variance of the fixed effects is at least as large in democracies as in autocracies. While there is more total variability in growth rates in autocracies, they contend that the amount of variation attributable to leaders is higher in democracies.

There is also interest in the effectiveness of well-compensated business leaders. To investigate the effects of CEOs on the success of their firms, many researchers have conducted variance decompositions, which use regressions to estimate how much variance in an outcome of interest is explained by adding CEO-specific variables [see (*10*) for a review]. These studies typically report large effects, but for reasons we discuss below, these studies are likely to overstate the importance of CEOs.

In the realm of sports, analytics is becoming more prevalent and influential, but most of the analysis is devoted to player performance rather than coaching and leadership. Several studies have investigated the effects of coaching transitions, typically finding small effects [e.g., (*11*)]. Other studies use methods similar to the variance decomposition approach in the CEO literature and find that coaches explain a meaningful share of variation in the success of their teams [e.g., (*12*)].

## MATERIALS AND METHODS

Our goal is to provide a general test of whether leaders matter for particular outcomes of interest. As with the previous literature, we cannot estimate the effects of any individual leader with statistical confidence, but we can ask whether leaders matter in the aggregate. Are some leaders better than others, such that we can statistically reject the null hypothesis that all leaders are the same with respect for a particular outcome? After introducing our method, we compare it with other methods used in the prior literature and explain how it can improve upon or complement them.

There are several methodological challenges associated with estimating leader effects. A key challenge, and the primary focus of this paper, is inference. Suppose that we observe some apparent correlation between leaders and outcomes as in the examples discussed in Introduction. We would expect some of that apparent correlation just by chance, and we would like to account for the idiosyncrasies of luck to determine whether that correlation is indeed statistically significant. As we will show, because of random noise and serial correlation in leaders and outcomes, existing methods often fail to distinguish leadership from luck.

Another set of challenges has to do with identification. If we detect a statistically significant relationship between leaders and outcomes, then it might be attributable to leader effects, or there might be other reasons that outcomes systematically correspond with leaders. For example, if the outcome of interest affects leader turnover, then this could also generate a correlation between leaders and outcomes. Although we cannot entirely remove these concerns, we can show through simulations that the substantive relevance of these identification concerns is minimal in the settings we study.

Our general strategy involves regressing an outcome on leader fixed effects, recording a summary statistic of fit, and then simulating the distribution of summary statistics that we would expect under the null. As summary statistics of fit, the *r*^2^, adjusted *r*^2^, and *F* statistic will all produce identical *P* values and implied effect sizes in our subsequent analyses because, for a given sample size and number of regressors, these statistics all increase monotonically as the others increase. For the purposes of this paper, we focus on the *r*^2^ statistic, which is familiar to social scientists and has a substantive interpretation as the proportion of variation in the outcome that appears to be explained by the leader fixed effects. However, if subsequent practitioners would prefer using another fit statistic, then that is perfectly allowable within our framework.

In and of itself, the *r*^2^ statistic is not particularly informative. A high value could reflect leader effects, but it could also reflect within-unit variation over time unrelated to leader effects, or it could suggest that the regression with many independent variables overfit random variation in the outcome. Therefore, we need a strategy for simulating the distribution of *r*^2^ statistics that we would expect under the null hypothesis of no leader effects. To do this, we randomly permute the ordering of leaders within each unit, keeping the tenure of each leader the same as in the real data but varying the order in which each leader served. For each random permutation, we again regress growth on leader fixed effects and record the *r*^2^ statistic. We repeat this procedure many times to estimate the distribution of *r*^2^ statistics we would obtain under the null of no leader effects. The proportion of random permutations that produce an *r*^2^ statistic greater than that from the real data is an estimated *P* value, testing the null hypothesis that all leaders are equally effective with respect to this particular outcome.

These hypothesis tests are one sided because genuine leader effects can only increase the real *r*^2^ statistic relative to the expected *r*^2^ statistic under the null, and there will never be a situation where we would expect that the real *r*^2^ to be lower than the average simulated *r*^2^. In some cases, of course, the real *r*^2^ statistic can turn out to be smaller than the average simulated *r*^2^, but this would not constitute any evidence of leader effects. If there are no leader effects, then the real *r*^2^ statistic will be larger than the simulated average half the time, and the other half the time, it will be smaller.

Before implementing our method, we typically take several steps to prepare the data for analysis and improve statistical precision. These steps are optional and should likely vary depending on the substantive setting being studied. For example, we can demean the outcome by year to remove consistent time trends across units. We could also demean by unit but this would have no impact on our subsequent *P* values. Our inferential strategy implicitly accounts for variation across units. We might also want to partial out the effects of other factors that are unrelated to the leader effects of interest. For example, when studying sports coaches, we partial out measures of opponent quality and home field advantage. If there are any observations with missing data, then we drop those observations and stitch together blocks of time, coding a new time variable so that we have a single, contiguous stretch of time with complete data for each unit. This step allows us to take advantage of all the observations with nonmissing values when permuting the data.

Having processed the data, we regress our outcome of interest on leader fixed effects and record the *r*^2^ statistic. Then, to simulate the distribution of *r*^2^ statistics that we would expect under the null, we randomly permute the leader identifiers, keeping each leader stint together as a block in each permutation, rerun the regression of the outcome on leader fixed effects, and repeat this procedure many times.

The summary of our procedure is as follows:

1) Regress the outcome on leader fixed effects and record the *r*^2^ statistic.
2) Randomly permute leaders within each unit, sampling each leader stint as a block.
3) Regress the outcome on the permuted leader fixed effects and record the *r*^2^ statistic.
4) Repeat steps 2 and 3 many times, recording the proportion of cases where the *r*^2^ from the permuted data is greater than that from the real data.

Our procedure is closely related to many others using some form of permutation test or randomization inference [e.g., (*13*–*16*)]. One distinction between our approach and a canonical application is that we are not estimating a treatment effect directly. Instead, we use permutation tests to estimate the distribution of *r*^2^ statistics that we would expect under a null hypothesis of no leader effects. Our approach is also closely related to methods for testing hypotheses about multiple treatments [e.g., (*17*), pp. 963 to 967]. One could think of each leader as a separate treatment, where we want to test whether the treatment effects are jointly distinguishable from one another. However, rather than using conventional approaches, we must use blocked randomization inference to account for time trends and serial correlation.

The logic of our random permutation tests is as follows. Assume that leader transitions are unrelated to potential outcomes, such that in the absence of any leader effects, there should be no systematic correspondence between leader indicators and outcomes. There are three ways we can get a high *r*^2^ statistic when we regress growth on leader indicators. First, there could be leader effects, and this is what we would like to identify. Second, there could be serial correlation or genuine trends in an outcome over time within units even in the absence of leader effects, and the leaders who happened to serve in good (or bad) times will get credit for this in the regression. Third, the leader fixed effects could be overfit to random, year-to-year fluctuations in the outcome or even measurement error, further inflating the *r*^2^ statistic. Therefore, to test for leader effects, we would like our random permutation tests to incorporate the last two factors but not (all of) the first.

In our random permutations, the number of fixed effects in each regression is held constant, and the distribution of tenure across leaders is also held constant. This means that the extent of overfitting is the same, in expectation, in the real data and the permuted data. This assumes that the researcher does not use the observed data to make specification choices or conduct multiple tests and selectively report the significant ones. If a careless researcher modified the above procedure to better fit the observed data or selectively reported the results of multiple tests, then the resulting *P* values would be misleading. This is, of course, a concern with virtually all quantitative analyses, although we attempt to mitigate these concerns in this case by specifying a simple and generalizable procedure that will be applied in the same way to different datasets.

Furthermore, although serial correlation or unit-specific time trends unrelated to leaders will inflate the *r*^2^ in the real regression, they should inflate the *r*^2^ from the permuted regressions to the same degree. However, if there are genuine leader effects, then this will increase the *r*^2^ in the real regression by more than it increases the *r*^2^ in the permuted regressions. Therefore, if the *r*^2^ from the real data is larger than that from the random permutations, then this is an indication that variation in the outcome coincides with the intervals of time in which different leaders served, suggesting that some portion of that *r*^2^ statistic can be attributed to leader effects rather than just serial correlation or chance.

Our practice of sampling each leader as a block and maintaining the same distribution of contiguous periods of service in our permutations is important. To our knowledge, the approach in the literature closest to our own is a robustness check from Yao and Zhang [(*8*), p. 420]. However, instead of sampling each leader as a block, Yao and Zhang (*8*) sample each year independently such that leaders’ terms are no longer contiguous in the random permutations. This approach accounts for the possibility of overfitting discussed above, but it does not account for the possibility of serial correlation or unit-specific time trends, and as a result, this test is likely to reject the null even if there are no leader effects.

To understand how our method performs in different scenarios, let us consider how various features of the data generating process will influence the *r*^2^ statistic in the real and permuted datasets. Recall that all of the regressions run under RIFLE will include a set of leader fixed effects. By definition, $r^{2}\equiv 1-\frac{\text{RSS}}{\text{TSS}}$, where RSS is the residual sum of squares and TSS is the total sum of squares. In our regressions, the TSS is identical for both the real data and the permuted data where the ordering of leaders is randomly shuffled. Therefore, to think about how our method works, we need to think about how leader effects, time effects, and random noise influence the RSS. Random noise increases the RSS, and it increases the RSS in the same way, in expectation, in the real dataset and the permuted datasets. If the noise was expected to affect the RSS differently in the real data, then it would not be random. Similarly, time effects that are unrelated to leaders’ tenures will also increase the RSS, and they will increase the RSS the same way, in expectation, in the real and the permuted datasets. This is why our method of permuting leader’s tenures accounts for noise and time trends unrelated to leaders.

How do leader effects influence the RSS? A constant effect for each leader should have no effect on the RSS in the real dataset. In this context, $\text{RSS}\equiv \sum_{i}\sum_{t}{\left(Y_{\mathrm{it}}-{\bar{Y}}_{i}\right)}^{2}$, where *i* denotes leaders and *t* denotes observations within each leader. That is, the RSS is the sum of squared deviations of each data point from the mean for each leader. A constant effect for each leader would mean that the outcome is shifted by the same amount for all observations of each leader, such that each *Y_it_* would be shifted by the same amount as each ${\bar{Y}}_{i}$ and the RSS would be unchanged by leader effects. However, in the permuted datasets, leader effects would increase the RSS. For each permuted leader’s tenure that overlaps with multiple actual leader’s tenures, leader effects will shift observations by different amounts within each permuted leader, thereby increasing the RSS. This means that in the presence of genuine leader effects, we expect the RSS to be lower for the real dataset than in the permuted datasets, meaning that the *r*^2^ will be higher.

To fix ideas, consider the simplest possible example where our test would allow us to say something about leader effects. Suppose that there is one unit with two leaders and three periods. Without loss of generality, suppose that leader A served during the first two periods and leader B served during the last period. In this simple example, there are only two ways to permute the leaders. We can assign leader A to the first two periods, as in the real world, or we can assign her to the last two periods. If leader A is better than leader B, or vice versa, then we would expect the outcome from the first two periods to be more similar to each other than they are to the value from the third period, and the real data will give a higher *r*^2^ statistic. If there are no leader effects but there is random noise or serial correlation, then either permutation is equally likely to give a higher *r*^2^.

This simple example illustrates several features and limitations our approach. First, identification comes from leaders who serve different periods of time. If there were four periods and each leader served two periods, then both permutations would yield the same *r*^2^. Next, our procedure behaves poorly when there are few leaders. In the example above, the *P* value can only take one of two possible values, but asymptotic refinement improves quickly with more units or more leaders per unit, so long as there is variation in lengths of service. Furthermore, our procedure requires that we put some structure on the timing of leader effects. Suppose that a leader’s actions affect the outcome in the next year. In that case, we would have no opportunity to detect leader effects in the simple example above. For our subsequent analyses, we will assume that leaders only affect outcomes in the years in which they serve, but one could easily conduct separate tests where they assume that these effects are lagged by 1 year, 2 years, etc. Last, our approach does not require us to hypothesize that one particular leader is better than another. For the purposes of this study, we are agnostic about which leaders are better. We test whether some leaders are different from others in ways that matter for various outcomes of interest.

Our method is not robust to endogenous turnover decisions in the absence of leader effects. To see the issue, suppose that leaders do not matter for the outcome in question but voters or employers behave as if they do, retaining leaders after good outcomes but replacing them after bad outcomes. This behavior would be a source of bias for RIFLE and other methods of estimating leader effects. To address this issue, we estimate the actual relationship between performance and turnover for politicians, CEOs, and coaches. We then provide simulations showing that the bias due to endogenous turnover is likely to be small in the settings we study. The same approach can be used to evaluate the extent to which endogenous turnover is a source of bias in other settings.

RIFLE is a general, flexible method that can be applied to any setting with an objective outcome of interest and leaders who serve different periods of time. We have developed a Stata package that will allow future researchers to easily apply this method to many different contexts and outcomes to better understand where, when, and why leaders matter. The package can be downloaded by typing “ssc install rifle” within Stata.

RIFLE relates to methods used in other papers that rely on leader fixed effects in one way or another to study political leaders (*9*), CEOs [e.g., (*18*)], and even teachers (*19*). These studies typically use parametric inferential strategies that, as we show, tend to produce misleading results, but our approach to inference is different. We do not assume that the *r*^2^ statistic, or a related summary of fit, from a set of leader-specific fixed effects will be zero when leaders do not actually affect the outcome. Because of random noise in the data, serial correlation, and unit-specific trends, leader fixed effects will increase the *r*^2^ even when leaders do not matter. Using the adjusted *r*^2^, as Bertrand and Schoar (*18*) do, partially accounts for the problem of overfitting to random noise, but it does not address time trends and serial correlation. Fitza (*10*) points out that previous studies overestimate leader effects by fitting random noise and proposes that researchers correct for this by conducting a placebo test with randomly generated outcome data. While this approach is useful, it also does not correct for time trends or serial correlation. RIFLE accounts for these factors without requiring additional assumptions about the nature of the serial correlation or the unit-level trends. In this sense, our approach is more conservative and will be less prone to detecting leader effects in cases where there are none.

A preview of one of our results illustrates the idea that the *r*^2^ or adjusted *r*^2^ statistics alone are insufficient for assessing leader effects. In a subsequent analysis, we test whether U.S. mayors affect changes in employment in their cities. When we randomly permute the terms of service for mayors, there should be no leader effects in these permuted datasets because terms of service are random. Nonetheless, the average *r*^2^ statistic from our random permutations in this setting is 0.606, meaning 61% of the variation in employment across cities appears to be explained by leader fixed effects, although the leader variables correspond to random time intervals. The adjusted *r*^2^, which is designed to mitigate overfitting, is still 0.525. If we first demean by city, then the *r*^2^ and adjusted *r*^2^ statistics are 0.343 and 0.209, respectively. None of these statistics can separate leader effects from other forces because the numbers are large even when there are no leader effects. Whenever there is serial correlation in the outcome or there are unit-specific time trends unrelated to leaders, we would expect a large *r*^2^ statistics regardless of whether leaders matter. As we will see, although the *r*^2^ and adjusted *r*^2^ statistics are large when we regress city employment on mayor fixed effects, RIFLE shows that they are no larger than we would expect by chance if mayors do not affect employment.

RIFLE also offers some advantages relative to the method of Jones and Olken (*6*). They use a similarly nonparametric inferential approach, comparing the changes in economic growth in periods with leadership transitions to the distribution of changes in periods when there are no transitions. However, their analysis only includes leader transitions arising from unexpected deaths, i.e., those due to an accident or illness in office. While unexpected deaths provide plausibly exogenous changes in leadership, identification comes from a relatively small number of leader transitions. Specifically, they have only 57 such unexpected transitions from a panel of 130 countries since 1945. Our approach provides more statistical precision using more data.

One concern with focusing on unexpected leader transitions is that the results may reflect the disruptive effects of an unanticipated transition of power. To the extent that unexpectedly changing leaders disrupts the government or economy, this will be reflected in estimates of leader effects using this strategy. While Jones and Olken (*6*) take measures to reduce the possibility that disruption contaminates their results, such as excluding the first year or two years after the transition, some concerns remain. Besley *et al.* (*7*) show that the average change in growth following an unexpected leader transition is negative. In particular, they show that there is a 0.2–percentage point reduction in annual growth during the 5 years after an unexpected transition in leadership. Unless there are disruptive effects, it is hard to see why the average effect of random leader transitions should be predictability negative.

RIFLE also offers practical advantages in terms of its generalizability. The method of Jones and Olken (*6*) requires identifying not only leader transitions due to death in office but also knowledge of the cause of the leader’s death. While uncovering such information may be feasible for world leaders, it may be impractical or impossible in the case of other leaders serving in less prominent positions. Even for the large U.S. cities that we study in this paper, we sometimes had difficulty finding the names of the mayors who served in the past. We suspect that finding detailed information about their cause of death would require heroic effort. Furthermore, there are many settings for which unexpected deaths in office are extremely rare. Fortunately, our method does not require additional information beyond the leaders’ terms of service and so should be more applicable to studying a wide range of leaders.

We also note some limitations of our method, which are shared with others in the literature. We can think of our approach as testing the null hypothesis that all leaders are equal with respect to a particular outcome of interest. We conclude that leaders matter when we can reject the hypothesis that all leaders are equal. One reason that we might fail to reject the null is if selection reduces the variation among those who become leaders. For instance, electoral competition or rigorous hiring practices may result in only high-quality leaders serving. If these leaders perform equally well, then there will be no leader effects according to our definition. However, such a null finding would not imply that replacing a sitting leader with a random person would have no effects. Furthermore, simply rejecting the null might not be particularly interesting in some cases. As with many other examples of hypothesis testing, we might put little probability mass on the null; of course, some leaders are different from others. However, as we discuss below, we can also use this method to say something about substantive effect sizes and assess the extent of variation attributable to leader effects. Last, as noted above, RIFLE will be biased when leaders do not matter but voters or employers behave as if they do, a phenomenon we label endogenous turnover.

Some empirical studies ask about the effects of selecting one type of leader or another. For example, in the context of U.S. mayors, there are studies on the effect of Democrats versus Republicans (*20*–*22*), females versus males (*23*), and black versus nonblack mayors (*24*). These kinds of studies can, in principle, demonstrate that leaders matter and they can further explore which dimensions of leaders’ characteristics or backgrounds matter most for particular outcomes. However, this literature has produced a lot of null results, and even when results are not null, we might worry that many hypotheses were tested across many characteristics of leaders and many outcomes, leading us to wonder whether and how much variation in leaders really matters for a particular outcome of interest. In these cases, a more general test of leader effects is warranted. We see our method as a complement to (but not a replacement for) design-based studies of the effects of particular kinds of leaders. Iteration and interplay between our general test of leader effects and more specific testing of particular dimensions of leadership could be particularly fruitful in settings where leader effects have previously been elusive.

To assess the properties of RIFLE, we have conducted a series of Monte Carlo simulations. First, we show that if leaders and outcomes are not related, then we will not detect leader effects. Under the null hypothesis, *P* values should be uniformly distributed between 0 and 1, and this is exactly what we find. We simulate datasets with a certain number of units and time periods. For our initial simulations, we assume that each leader’s tenure is randomly drawn uniformly from integers between 1 and 5. We simulate an extra five periods for each unit and remove the first five periods. This ensures that the simulated dataset starts in the middle of some leaders’ tenures, making the simulations more similar to our subsequent analyses with real data. The outcome in the first period is drawn independently from a standard normal distribution, and the outcome in each subsequent period is a weighted average of the outcome in the previous period and a new draw from a standard normal distribution. This means that there is random variation in the outcome from year to year and there is also serial correlation over time within each unit, but the outcome is unrelated to leaders.

**Fig. 1:**
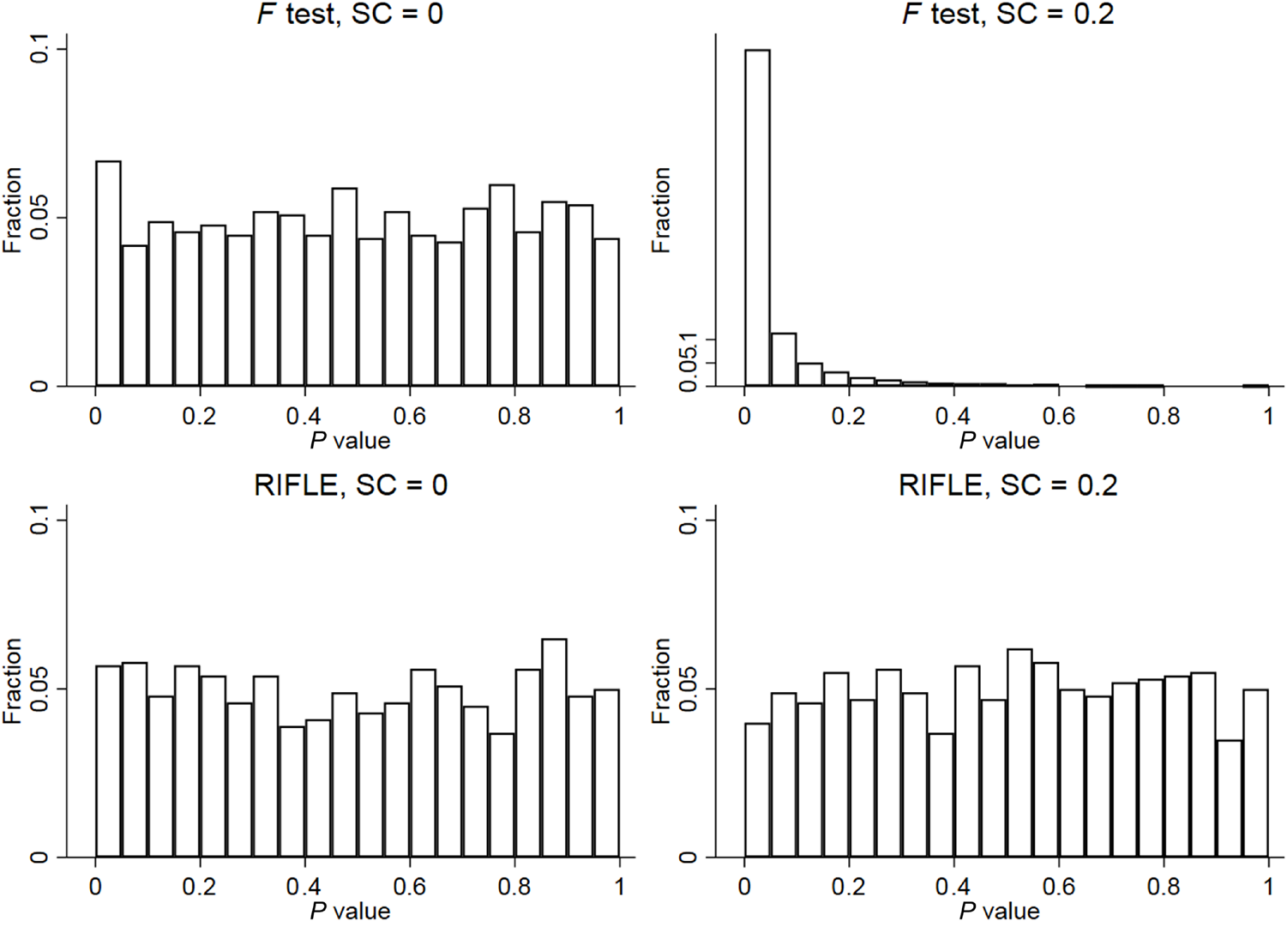
Monte Carlo simulations with random noise, serial correlation, but no leader effects. Each histogram shows the distribution of *P* values resulting from 1000 simulated datasets with serial correlation (SC) of different magnitudes. The top row presents results from a standard *F* test, and the bottom row presents tests using RIFLE.

In Fig. 1, we present results from simulations with 20 units and 20 periods per unit, although results are similar even when we have as few as 5 units and 5 periods. We vary the extent of serial correlation in the data by varying the weight of the previous period’s outcome in the current period’s outcome, and we show results for weights of 0, i.e., no serial correlation, and 0.2, i.e., modest serial correlation. For each level of serial correlation, we simulate 1000 datasets and implement RIFLE. Furthermore, to compare RIFLE to a more standard method, we also implement an *F* test of joint significance after running a regression of the outcome on leader fixed effects.

The top row of Fig. 1 shows the results of the *F* tests, and the bottom row shows the results of RIFLE. When there is no serial correlation, both RIFLE and the *F* test perform well, producing a uniform distribution of *P* values as we should expect when there are no leader effects. When we introduce serial correlation, however, RIFLE continues to perform well, while the *F* test markedly over-rejects the null. These simulations confirm that random noise and serial correlation do not contaminate the results of RIFLE as they do for more standard methods. Furthermore, our *P* values are reliable even with a small number of units and periods. In addition, notice that our procedure never requires the researcher to specify the nature of serial correlation in the data. If growth is unrelated to leaders’ tenures, then our test will not wrongly detect leader effects.

What if all leaders are equally able but there is a transition cost associated with political turnover? This is an important question because transition costs are a key concern for the methodology of Jones and Olken (*6*). Because they focus on periods right around a political transition, these transition costs could lead them to overstate leader effects. To assess the effect of transition costs for our method and that of Jones and Olken (*6*), we implement another battery of Monte Carlo simulations, shown in the Supplementary Materials. As expected, transition costs strongly bias the test of Jones and Olken (*6*) test against the null, while the implications of transition costs for RIFLE are much smaller. For transition costs to meaningfully bias our approach, these costs have to be very large, and in these cases, the bias is in the opposite direction, meaning that we under-reject the null. While transition costs would produce a false positive with the methodology of Jones and Olken (*6*), they lead our test to be conservative. The intuition is that every leader experiences exactly one transition cost during their tenure, so the estimated leader coefficients end up being similar in the real data and the *r*^2^ is low. When we randomly permute the leader’s tenures but keep the outcome data the same, some leaders end up with multiple transition costs in their tenure, and others have none, which spreads out the estimated leader coefficients and increases the *r*^2^. However, again, for reasonably sized transition costs, the implications for our test are minimal.

We have seen that RIFLE performs reasonably well when there are no leader effects. However, if there are genuine leader effects, then will it reliably detect them? The value of our method will be limited if there is not sufficient statistical power to detect meaningful effects when they do exist. Without having any specific hypotheses about which leaders are better, we do not know how we could achieve greater statistical power without over-rejecting under the null. As an example, our test should provide more power than that of Jones and Olken (*6*) because instead of using only data from years around exogenous transitions, we are using all years and all leaders where data are available. Furthermore, the *r*^2^ is an efficient statistic for our purposes because it implicitly puts more weight on the leader coefficients that are more precisely estimated, i.e., where there are more periods per leader. We have also explored alternative statistics such as the SD of the estimated leader coefficients, but these statistics are less efficient than the *r*^2^ because many of those coefficients are imprecisely estimated. That is, we have designed our test with the goal of obtaining reliable *P* values while also maximizing statistical power.

**Fig. 2:**
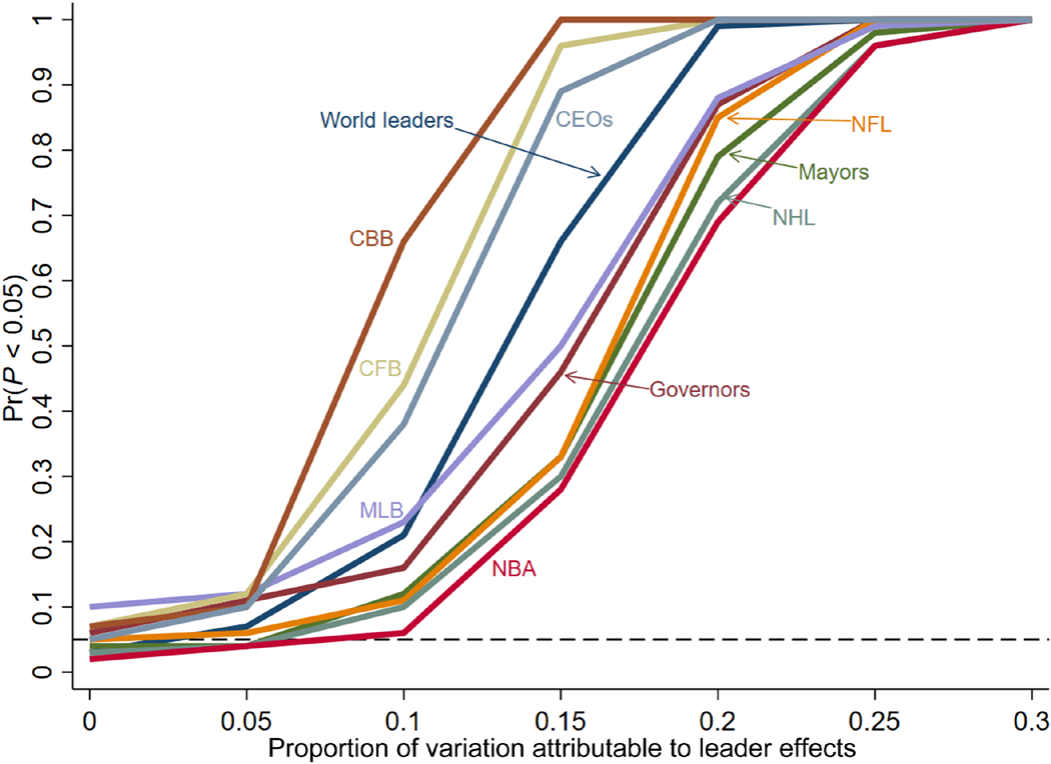
Statistical power across settings. The figure shows the simulated probability of rejecting the null for different effect sizes for each of the settings analyzed. CBB, college basketball; CFB, college football.

In Fig. 2, we show the results of Monte Carlo simulations designed to explicitly assess the statistical power of our approach in different datasets. For each of the datasets that we subsequently analyze, we run simulations in which we replace the actual outcomes with simulated data with known leader effects. Specifically, for each simulated dataset, we generate a random variable from a standard normal distribution for each leader that indicates their individual effect. Then, for each observation in the dataset, the outcome is simulated as random noise, drawn from a standard normal distribution, plus the leader effect that is multiplied by a number, which corresponds to a particular magnitude of leader effects. For instance, when the leader effects are multiplied by one-ninth, this means that leader effects explain ^1^/_10_ of the variation in the outcome variable. For each simulated dataset, we generate 19 permutations, corresponding to 20 different possible *P* values, and we record whether the *P* value is less than 0.05. Then, for each magnitude, we repeat this procedure 100 times and record how often we reject the null. Figure 2 plots the results of these simulations for all settings and for magnitudes of 0, 0.05, 0.1, 0.15, 0.2, and 0.3.

As expected, when there are no leader effects, our test rejects the null about 5% of the time, as it should. When leader effects are small, say 5% of the variation in the outcome, our test will not reliably detect them in these settings. However, once leader effects increase to 15%, our test reliably detects them in most settings. In addition, if leader effects explain 25% of the variation in the outcome or more, then our test is virtually guaranteed to reject the null in all of our datasets. As expected, our power varies depending on the amount of data available. We have the most statistical power for our data on college basketball coaches, and we have less power for National Hockey League (NHL) coaches, National Basketball Association (NBA) coaches, and U.S. mayors.

If our test does lead us to reject the null, then we have evidence that leaders matter, but that alone tells us nothing about the substantive size of leader effects. For many questions in the social sciences, including this one, our prior beliefs put little mass on the null hypothesis that an effect is exactly zero, and we care more about substantive significance than statistical significance (*25*). As we have discussed, the *r*^2^ statistic itself is not substantively interpretable. It tells us the proportion of variation in the data apparently explained by leader fixed effects, but that number includes leader effects plus unit-specific time trends and the overfitting of random noise. However, the difference between the real *r*^2^ and the average of the permuted *r*^2^ statistics should increase with leader effects, so that difference should be useful for interpreting effect sizes. Even that number, however, does not have a direct substantive interpretation. One reason is that genuine leader effects should increase both the real *r*^2^ and the permuted *r*^2^, although they should increase the former more than the latter. Therefore, if *r*^2^ were greater in the real data than in the permuted data and one interpreted that difference as the proportion of variation attributable to leader effects, then they would understate the true effect. Therefore, to say more about effect sizes, we recorded this difference from the power simulations above.

**Fig. 3:**
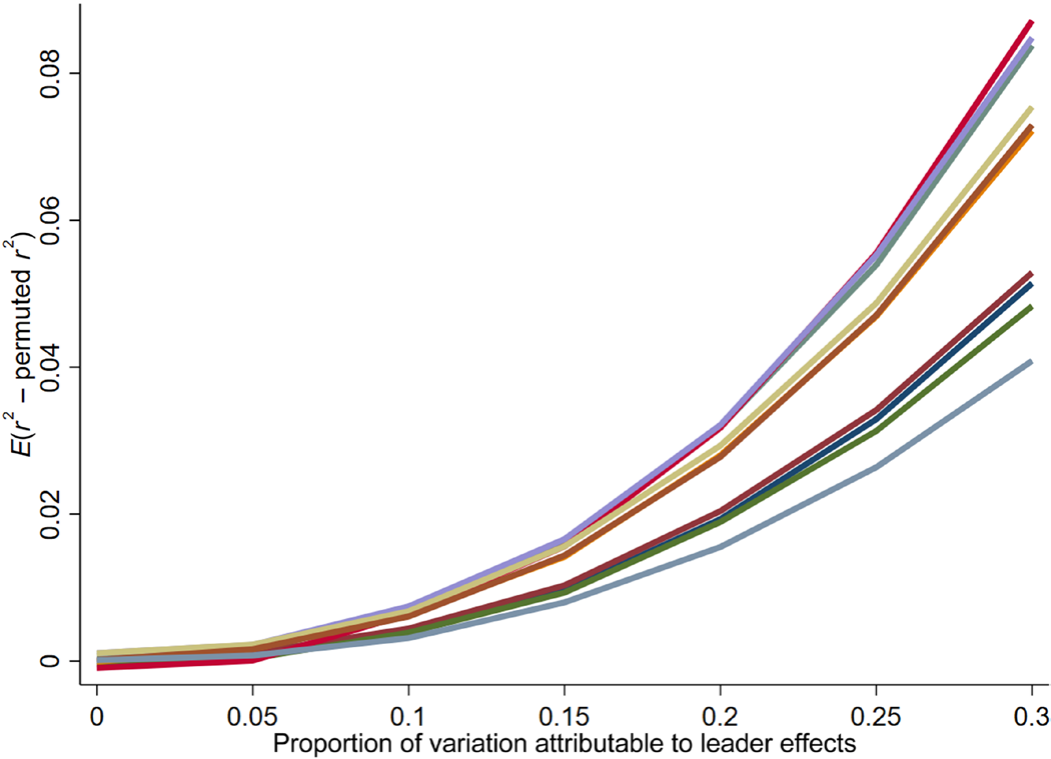
Interpreting effects sizes across settings. The figure plots the expected *r*^2^ minus the average permuted *r*^2^ for different effect sizes and settings. We later use these simulation results to assess substantive effect sizes.

Figure 3 shows the average difference between the real and permuted *r*^2^ statistics for each of the settings in our subsequent analyses and for different magnitudes of leader effects ranging from 0 to 30%. As expected, there is not a one-to-one relationship between the difference in *r*^2^ and the actual proportion of variation attributable to leader effects. Instead, the relationship is nonlinear, and, as expected, the average slope is less than 1. The color coding is the same as in Fig. 2, and we see that the slope tends to be lower when statistical power is lower. These simulation results, in conjunction with our actual results using real data, can be used to assess effect sizes. For example, suppose that when we analyze the effects of U.S. mayors on employment, examining data from 1970 to 2015, we obtain an *r*^2^ that is 0.01 greater than the average *r*^2^ from the random permutations, which would imply that mayor effects explain more than 15% of the within-city variation in employment. For any subsequent results that do imply leader effects, we can use this approach to interpreting the substantive size of those effects.

For our results below, whenever we obtain a positive difference between the real *r*^2^ and the permuted *r*^2^, we will estimate the effect size most consistent with the observed difference using linear interpolation. Specifically, we will use the simulation results shown in Fig. 3, determine the interval of true effect sizes where we would expect an observed difference of this magnitude, run a linear regression of effect size on the difference in *r*^2^ for the two data points flanking that interval, and then compute the predicted value for the actual observed difference. This number can be interpreted as a rough estimate of the proportion of variation attributable to variation in leader abilities, and it is reported in the “prop” column of our results tables below.

What if leaders do not matter for a particular outcome but their terms of service are influenced by that outcome, perhaps because voters or employers believe leaders matter? In general, when leaders’ chances of being replaced are influenced by the outcome of interest, this poses a problem for our test, and the bias can go in either direction. We refer to such a phenomenon as endogenous turnover, and we explore its implications for RIFLE with a theoretical model in the Supplementary Materials. To assess its empirical implications in the settings we study in this paper, we conduct two related analyses. First, we examine the actual degree of endogenous turnover by regressing leader turnover against lagged outcomes in each of our settings. Second, we run Monte Carlo simulations to evaluate the amount and direction of bias given the empirically observed correlation between turnover and outcomes in each of our settings. The results of both analyses are shown in Table 1.

**Table 1:** Simulated FRR with endogenous turnover. FRR, false rejection rate; SG&A, selling, general, and administrative expense; ROA, return on assets.

**Setting**	**Outcome/sample**	**Avg. turn**	**Slope**	**Δ*r*^2^**	**Serial correlation**	**FRR**
World leaders	All	0.192	−0.024	0.003	0.215	0.055
	Autocracies	0.055	−0.006	0.001	0.295	0.035
	Democracies	0.269	−0.027	0.003	0.329	0.045
	Transitional	0.196	−0.028	0.004	0.152	0.035
Governors	Income	0.200	−0.003	0.000	−0.178	0.050
	Employment	0.200	−0.003	0.000	0.656	0.040
	Revenue	0.200	0.009	0.000	−0.094	0.055
	Expenditures	0.200	−0.003	0.000	−0.004	0.035
	Federal aid	0.200	0.016	0.001	−0.229	0.060
	Property crime	0.200	−0.012	0.000	−0.026	0.050
	Violent crime	0.200	−0.015	0.001	−0.053	0.035
Mayors	Income	0.163	−0.003	0.000	0.151	0.060
	Employment	0.163	−0.004	0.000	0.719	0.060
	Public employment	0.163	−0.005	0.000	−0.064	0.070
	Public salary	0.163	0.005	0.000	−0.253	0.070
	Property crime	0.163	0.007	0.000	−0.022	0.060
	Violent crime	0.163	0.007	0.000	−0.017	0.060
CEOs	Cash flow	0.114	0.008	0.000	0.825	0.050
	Investment	0.114	−0.005	0.000	0.800	0.055
	Cash holdings	0.114	0.003	0.000	0.855	0.050
	Interest coverage	0.114	−0.002	0.000	0.238	0.045
	Leverage	0.114	0.002	0.000	0.033	0.035
	Advertising	0.114	−0.015	0.000	0.944	0.035
	SG&A	0.114	0.005	0.000	0.122	0.040
	ROA	0.114	−0.005	0.000	0.171	0.035
	Operating ROA	0.114	−0.015	0.001	0.687	0.055
MLB	Scored	0.313	−0.094	0.038	0.456	0.065
	Allowed	0.313	0.097	0.038	0.382	0.055
	Margin	0.313	−0.124	0.063	0.476	0.080
	Win	0.313	−0.130	0.074	0.502	0.080
NFL	Scored	0.241	−0.107	0.060	0.442	0.055
	Allowed	0.241	0.089	0.041	0.346	0.055
	Margin	0.241	−0.120	0.075	0.457	0.055
	Win	0.241	−0.133	0.092	0.416	0.045
CFB	Scored	0.226	−0.059	0.019	0.434	0.045
	Allowed	0.226	0.063	0.021	0.429	0.060
	Margin	0.226	−0.071	0.028	0.505	0.060
	Win	0.226	−0.071	0.028	0.417	0.065
NBA	Scored	0.306	−0.033	0.005	0.531	0.030
	Allowed	0.306	0.102	0.049	0.565	0.085
	Margin	0.306	−0.131	0.080	0.621	0.045
	Win	0.306	−0.134	0.085	0.603	0.060
CBB	Scored	0.156	−0.020	0.003	0.489	0.055
	Allowed	0.156	0.036	0.009	0.534	0.035
	Margin	0.156	−0.050	0.018	0.533	0.060
	Win	0.156	−0.052	0.019	0.464	0.065
NHL	Scored	0.364	−0.088	0.033	0.498	0.070
	Allowed	0.364	0.134	0.076	0.507	0.040
	Margin	0.364	−0.141	0.085	0.578	0.070
	Win	0.364	−0.145	0.089	0.543	0.065

The third column, Avg. turn, shows the average annual rate of leader turnover. For instance, the first row of the table shows that, on average across our sample, 19% of world leaders turn over each year. This rate varies across settings. For example, the turnover rate of world leaders is notably lower in autocracies (5.5%) than in democracies (27%). CEOs tend to have long tenures with an average annual turnover rate of only 11%, and on the other end of the spectrum, more than a third of NHL coaches (36%) are replaced each year, on average.

The next column, Slope, reports the coefficient from a regression of the turnover dummy against the outcome lagged by 1 year. The outcome variables are standardized so that the coefficient can be interpreted as the change in turnover probability associated with a one-SD change in the outcome. All of the regressions include unit and year fixed effects. The column, Δ*r*^2^ reports the increase in the *r*^2^ of the regression that comes from adding the lagged outcome variable, relative to a baseline model including only unit and time fixed effects. This can be interpreted as the proportion of within-unit variance in leader turnover that is explained by the outcome.

To illustrate, the first row shows that, for all world leaders, a one-SD increase in economic growth is associated with a 2.4–percentage point reduction in the probability of leader turnover in the following year. Relative to the baseline turnover rate of 19%, this suggests that a one-SD increase in economic growth reduces the probability of leader replacement by about 12.5%. The Δ*r*^2^ column shows that adding the outcome variable to the turnover regression increases the *r*^2^ by only 0.003, relative to the model with only unit and time fixed effects. For autocracies, the estimated slope and Δ*r*^2^ are even smaller.

Looking across the other settings and outcomes, we find that turnover for governors, mayors, and CEOs is only weakly related to the outcome variables under consideration. The change in *r*^2^ is effectively zero in all cases and the estimated coefficients are generally below 1 percentage point.

Endogenous turnover appears to be much more robust among sports coaches. Across all the sports we study, all of the coefficients are statistically significant and in the expected direction. The number of wins in a season, in particular, is a reliable predictor of coach turnover. One potential explanation for the extent of endogenous turnover in this setting is that sports coaches can essentially be fired at any time, while in many political systems, for example, there are only occasional, discrete opportunities to replace a leader. In the National Football League (NFL), for example, a one-SD increase in wins is associated with a 13–percentage point reduction in the probability of coach replacement in the following year. Relative to the baseline turnover rate of 24%, this represents a 54% reduction. Adding last-season wins boosts the *r*^2^ of the regression by 0.09. Most of the other sports yield similar results, although endogenous turnover is weaker in college basketball.

We suspect that the stronger connection between outcomes and turnover in sports, compared to business and government, is due to the clarity of the leaders’ objectives. Coaches are, above all, responsible for winning games, whereas political leaders have multifaceted responsibilities, any one of which in isolation may play only a limited role in voters’ evaluation of the incumbent.

That we find evidence of only weak endogenous turnover in politics may appear counter to conventional wisdom about the importance of the economy in influencing elections, a phenomenon dubbed economic voting [e.g., (*26*)]. However, it bears emphasizing that even in standard economic voting models, the *r*^2^ attributable to gross domestic product (GDP) growth is not especially high. For instance, in the United States, a regression of incumbent party presidential vote share against GDP growth delivers an *r*^2^ of 0.23 in our study period (*27*). Moreover, standard models use vote share as the dependent variable, which is measured only in election years. We use leader turnover as the dependent variable and include all years. However, strong the correlation may be in election years, it is essentially constrained to be zero in nonelection years, which limits the extent of endogenous turnover, at least in democracies. Moreover, term limits create leader transitions unrelated to economic performance, further weakening the possible extent of endogenous turnover in practice.

Overall, we find relatively little evidence of endogenous turnover, except in sports. To further assess the implications of endogenous turnover for our substantive applications, we have conducted separate Monte Carlo simulations for each kind of leader and each outcome examined in our study. We start with the actual datasets used to generate our main results, keeping the number of time periods and units as they are. We simulate the outcome of interest and the leader identifiers according to a known process. Specifically, the outcome is drawn from a normal distribution irrespective of leaders but with serial correlation. We estimate the actual amount of serial correlation in each setting by regressing the outcome at time *t* against the outcome at time *t* – 1. The coefficients from these regressions are listed in the column, Serial corr., in Table 1. In our simulations, the outcome in each period is a weighted combination of the outcome in the previous period and a new random draw. The weight given to previous performance is based on the actual serial correlation of outcomes observed for the setting in question.

Although leaders do not matter in these simulations, leader turnover is probabilistically affected by the outcome in the preceding period. We set the average probability of turnover in each period equal to the average turnover propensity in the data (column Avg. turn in Table 1). We also allow the probability of turnover to vary according to the degree of endogenous turnover observed in the data. Specifically, we apply the coefficient from the Slope column of Table 1 to the simulated data. For example, in the world leader simulation, the simulated outcome data are multiplied by the coefficient of −0.024, meaning that when the outcome is one SD above average, the leader is 2.4 percentage points more likely to be retained. We use the actual coefficient from each setting in its corresponding simulation, so the extent of endogenous turnover in our simulations is determined by the actual extent of endogenous turnover in each setting.

Using these simulated datasets, we implement RIFLE to see if we reject the null hypothesis (i.e., *P* < 0.05) when there is endogenous turnover. We repeat this many times to estimate the false rejection rate (FRR) of our method assuming no leader effects but allowing for the level of endogenous turnover observed in that setting. Ideally, we would obtain an FRR of 0.05, and to the extent that our results deviate from that, we can learn the extent to which endogenous turnover leads us to over- or under-reject the null.

The column FRR in Table 1 reports the FRR for each setting. According to our Monte Carlo simulations, the rates are generally close to the ideal of 0.05. The highest FRR we observe is for points allowed in the NBA, where the FRR is 0.085. These results suggest that the endogenous turnover of leaders, to the extent actually observed in these settings, does not meaningfully bias our results.

The methodology of RIFLE is reasonably flexible and can be extended or modified in a number of ways. Suppose, for example, that a researcher has additional covariates and would like to use those in her analyses. We can think of several reasons to do this. One potential benefit of additional covariates would be statistical precision. If we have covariates that explain a lot of variation in the outcome of interest but are otherwise unrelated to leaders, then we could partial out the effects of these covariates in the preprocessing stage to improve statistical power. We recommend regressing the outcome on time fixed effects and these covariates, calculating the residuals, and then using these residuals as the outcome in the subsequent regressions.

Another common situation is one in which we have a hypothesis about variation in leader effects. Suppose, for example, that we expect leader effects to be greater in autocracies than democracies. The simplest way to proceed is to separately implement RIFLE for autocratic and democratic countries. If a country switches back and forth between autocracy and democracy, then there are several different ways to proceed. A researcher could split up the data within a unit, although this will often sacrifice power because identification comes from more leaders serving different periods of time. In our subsequent analyses of world leaders, we separately analyze autocracies, democracies, and countries that switch during our period of analysis.

A third motivation for using covariates involves delving into specific mechanisms and asking more subtle questions about the nature of leader effects. For example, in our subsequent analyses of U.S. governors, we find meaningful effects on public finance outcomes. A natural next question is whether these differences are explained by differences between Democratic and Republican priorities or if there are still meaningful differences between governors of the same party and from the same state. As before, a researcher could partial out the effects of a leader-specific covariate, such as party, in the preprocessing stage, although the effect in this case is changing the quantity being estimated rather than improving statistical power. Briefly, the same motivations for preprocessing and subsetting data in other settings can be imported to this setting as well.

## RESULTS

To demonstrate the substantive value of RIFLE, we estimate leader effects in several different settings and for several outcomes. We start with an analysis of world leaders and economic growth, a setting that has been rigorously studied previously, allowing us to benchmark our estimates against those from previous methods. Since Jones and Olken (*6*) already provide credible estimates of leader effects in this setting, with the small caveat that transition costs could potentially bias their estimates, it would be reassuring for us to recover similar estimates using RIFLE.

We use data on world leaders from Archigos (*28*) version 4.1. We use data on GDP by country and year from the Maddison Project (*29*). In this and all subsequent applications, our data indicate the identity of the leader holding each leadership position in each year. In cases where multiple leaders served in the same position in the same year, we record which leader held the position for the greatest portion of that year. We also code all outcomes of interest as proportionate growth from the previous year. This likely improves statistical power because we would expect good leaders to achieve better-than-average growth in the outcome of interest rather than immediately shifting the outcome to a different level, although there might be some substantive settings where a researcher will want to code the outcome as a level rather than a change.

Following the methodology described above, we analyze the effects of world leaders by regressing GDP growth, after demeaning the data by year, on a set of leader dummies. As shown in Table 2, the *r*^2^ from this regression is 0.275, and the average *r*^2^ from the permuted regressions is 0.244. The estimated *P* value from 1000 different permutations is 0.003, meaning that the *r*^2^ from the real data was larger than the *r*^2^ statistics from all but 3 of the 1000 permuted datasets. The difference between the real *r*^2^ and the average permutation of 0.031 implies that about 24% of the variation in economic growth within countries is attributable to variation in national leaders. In our dataset, the SD of growth, demeaned by country and year, is 6.0%. Therefore, as a country switches from an average leader to one that is one SD above the mean, they can expect GDP growth to be about 1.5 percentage points higher than normal. This is a substantively meaning number, and it is nearly identical to the substantive size of leader effects implied by the results of Jones and Olken [(*6*), p. 837].

**Table 2:** Results for political leaders. Avg, average permuted *r*^2^; Diff, the difference between the real *r*^2^ and the average permuted *r*^2^; Prop, the estimated proportion of the variation in an outcome explained by leader effects.

**Setting**	**Outcome**	**Years**	***r*^2^**	**Avg**	**Diff**	***P***	**Prop**
World leaders: all	GDP	1876–2010	0.275	0.244	0.031	0.003	0.242
Autocracies			0.176	0.149	0.027	0.053	0.227
Democracies			0.389	0.391	−0.002	0.506	0.000
Transitional			0.278	0.240	0.038	0.006	0.263
U.S. governors	Income	1930–2015	0.166	0.154	0.012	0.239	0.158
	Employment	1970–2015	0.450	0.501	−0.051	0.998	0.000
	Revenue	1951–2008	0.432	0.152	0.280	0.000	0.300
	Expenditures	1951–2008	0.206	0.186	0.020	0.069	0.196
	Federal aid	1951–2008	0.135	0.120	0.016	0.040	0.177
	Property crime	1961–2012	0.182	0.166	0.017	0.038	0.181
	Violent crime	1961–2012	0.173	0.157	0.016	0.084	0.177
U.S. mayors	Income	1970–2015	0.174	0.183	−0.010	0.770	0.000
	Employment	1970–2015	0.608	0.606	0.002	0.429	0.070
	Public employment	1970–2010	0.183	0.204	−0.022	0.624	0.000
	Public salary	1973–2010	0.098	0.108	−0.010	0.826	0.000
	Property crime	1986–2012	0.159	0.164	−0.005	0.718	0.000
	Violent crime	1986–2012	0.162	0.166	−0.005	0.690	0.000

We next estimate leader effects separately for democracies and autocracies, as classified by the Polity IV scores. Jones and Olken (*6*) classify countries according to their status at the time of a leader transition. Easterly and Pennings (*9*), on the other hand, classify countries according to their average scores over time. We divide countries into three categories—those that were always democracies, those that were always autocracies, and those that changed their status at least once over the period of study. Twenty-nine countries in our data were always autocracies, 35 were always democracies, and 89 transitioned at some point. We implement our method separately for each subset.

We find some evidence of leader effects in autocracies (*P* = 0.053), strong evidence of leader effects in transitional countries (*P* = 0.006), and little evidence in democracies (*P* = 0.506). We should not necessarily conclude that leaders matter more in transitional countries than autocracies, since we have more statistical power in the latter sample. The implied substantive sizes of leader effects are similar across both samples. For autocracies, the difference of 0.027 suggests that leader effects explain about 23% of variation in growth, and since the SD of within-country growth in autocracies is 7.4%, this implies that a leader who is one SD above the mean will increase GDP growth by about 2 percentage points more than an average leader. For transitional countries, the difference of 0.038 suggests that leader effects explain 26% of the variation in economic growth, and since the SD of within-country growth in transitional countries is 5.9%, this implies that a leader who is one SD above the mean will increase GDP growth by about 1.5%. Like Easterly and Pennings (*9*) but unlike Jones and Olken (*6*), we find that leader effects are not limited to autocratic nations, but the implied effect sizes are slightly larger for autocracies than transitional countries. Like Jones and Olken (*6*) but unlike Easterly and Pennings (*9*), we find little evidence for leader effects in democracies.

One potential interpretation of these results is that democracies more consistently select high-quality leaders, meaning that the differences between leaders are less meaningful. This is different from saying that the effect of a good leader is smaller in democracies. It could be that leadership matters in democracies, but good institutions make the differences between the leaders selected in equilibrium smaller. Regardless of the interpretation, we are reassured that RIFLE largely recovers the most credible existing estimates in the literature.

Also in Table 2, we extend our analysis to U.S. governors following the same basic methodology. In addition to analyzing economic outcomes, we also test for governor effects on public finance and crime. Data on U.S. state-level income per capita and employment come from the Regional Economic Information System (REIS) of Bureau of Economic Analysis. Data on state-level per capita property crime and violent crime come from the Federal Bureau of Investigation’s Uniform Crime Reporting Program, and public finance data on state-level revenues and expenditures come from the U.S. Census Bureau’s Census of Governments and Annual Surveys of Local Government Finances.

Our state-level economic data start in 1930 for personal income and in 1970 for employment. While these panels are shorter than those for world leaders, our power simulations suggest that we can still detect meaningful leader effects if they are present. However, there is little evidence of governor effects on income or employment.

Although we find no evidence that governors matter for aggregate economic outcomes, perhaps, governors differ in their use of various policy levers. To test for this, we examine state revenue excluding federal aid, state expenditures, and aid from the federal government to states from 1951 to 2008. Perhaps, some governors are better than others at securing federal aid, and perhaps, they raise and spend different amounts of money. We obtain positive point estimates for all three of these outcomes, and these estimates are statistically significant in the case of revenue and federal aid. The estimates imply that 18 to 20% of variation in these public finance outcomes can be explained by the abilities and priorities of individual governors. Although governors differ in their abilities or appetites to raise and spend money, those differences do not appear to translate into differences in state income and employment.

Perhaps, the primary effects of governors are outside the economy, in which case we should shift our focus to outcomes that are potentially shaped more directly through state-level actions. Pursuing this idea, we next examine the effects of governors on crime rates. Using data from 1961 to 2012, we analyze the effects of governors on property and violent crime rates. We find statistically significant governor effects on property crime rates (*P* = 0.038) and suggestive effects for violent crime (*P* = 0.084). The differences between the real *r*^2^ and the permuted *r*^2^ imply that governor effects explain about 18% of the variation in both property crime and violent crime.

To the extent that we detect governor effects for public finance and crime, are these effects simply explained by differences between Democrats and Republicans? We address this question by removing the average effects of party before implementing our procedure. Specifically, instead of simply demeaning by time, we regress our outcomes on state fixed effects, year fixed effects, and an indicator for the governor’s party, and we compute the residuals. When we do this, the estimated effects of governors on public finance and crime are virtually unchanged, suggesting that these effects are not explained by average differences between Democrats and Republicans. We estimate little average effect of party on these outcomes, suggesting that there must be meaningful variation in governors’ abilities and priorities within a given party and state. This exercise demonstrates the flexibility of our method and its ability to address additional questions and disentangle potential mechanisms driving leader effects.

We next study the effects of mayors for similar outcomes. We focus on the 100 largest U.S. cities according to 2015 population. For each city, we collected data on the names and service dates of mayors dating back at least to 1970, when our data on local income and employment begin. We have obtained complete data for 70 of the top 100 cities. Because annual income and employment estimates are not available by city, we match each city to its home county and use county-level income and employment data from REIS. We drop the two cities that are not the largest in their county (Long Beach, CA and Mesa, AZ). We can also weight the regressions according to the city’s share of county population, which is allowable within our methodology and has no impact on our subsequent results.

Can mayors reasonably be expected to affect economic growth in their cities? The existing literature offers differing perspectives. One school of thought argues that Tiebout (*30*) competition forces mayors to single-mindedly pursue economic development (*31*), with some going so far as to argue that cities are governed as “growth machines” that pursue development at the expense of all other priorities (*32*). Others contend that mayors are relatively weak executives that lack the power to control basic service delivery, much less to drive economic growth in their cities (*33*). We find little evidence of mayoral effects on income and employment in their counties, as shown in Table 2. In both cases, mayoral fixed effects appear to explain a substantial amount of the variation in the outcome, but we see that the placebo dummies explain just as much, on average. Similarly, when we examine public finance data on city employees and the average salary of city employees, we continue to find null results.

We next turn to an analysis of mayoral effects on crime, an outcome over which we might expect mayors to have greater influence. After all, mayors directly appoint police chiefs and shape law enforcement policy within their jurisdictions. Nevertheless, we find no evidence that mayors affect either property or violent crime rates in their cities. Our results reveal no evidence of mayoral effects for some of the most important outcomes in a city—the economy, the size of city government, and crime rates. These results are generally consistent with the argument that mayors simply lack control over governance and service provision within their jurisdictions [e.g., (*33*)]. Furthermore, we continue to obtain similarly null results when we focus exclusively on the cities that, according to surveys conducted by the International City Management Association, have a mayor-council system in which the mayor has more independent authority. Our null results, in this case, could be the result of insufficient data, but we fail to detect mayor effects even for the outcomes and cities where we would most expect them.

We also study CEOs of major U.S. companies between 1970 and 2015. Following Bertrand and Schoar (*18*), we obtain data on the identity of firm CEOs for 1970 to 1992 from the Forbes 800 files provided by K. J. Murphy and for subsequent years from Execucomp. We match the CEO data to firm-year outcomes of interest available from Compustat. After matching, we have data on the CEOs and outcomes of interest for an average of 395 firms each year and a total of nearly 600 unique firms over time. We focus on the nine outcomes of greatest interest in Bertrand and Schoar (*18*), and we follow their coding practices.

Table 3 shows the results. For all but one of the nine outcomes, our estimates are not statistically significant at the 0.05 level. Despite having high statistical power, more than for world leaders and almost as much as for college football coaches, our results are essentially null. Aside from the single outcome of cash holdings, we find no evidence that CEOs affect the performance of their firms.

**Table 3:** Results for CEOs, 1970–2016. Avg, average permuted *r*^2^; Diff, the difference between the real *r*^2^ and the average permuted *r*^2^; Prop, the estimated proportion of the variation in an outcome explained by leader effects.

**Outcome**	***r*^2^**	**Avg**	**Diff**	***P***	**Prop**
Cash flow	0.131	0.152	−0.021	0.681	0.000
Investment	0.126	0.136	−0.010	0.734	0.000
Cash holdings	0.169	0.148	0.021	0.030	0.224
Interest coverage	0.269	0.203	0.066	0.129	0.300
Leverage	0.164	0.221	−0.057	0.512	0.000
Advertising	0.177	0.179	−0.002	0.507	0.000
SG&A	0.572	0.253	0.319	0.060	0.300
ROA	0.136	0.147	−0.011	0.411	0.000
Operating ROA	0.147	0.168	−0.021	0.707	0.000

This result conflicts with much of the existing literature on CEOs, although as discussed above, the existing tests of CEO effects are prone to false positives because they do not account for serial correlation or firm-specific trends. When we use a more reliable approach to inference, we find little evidence that variation in CEOs explains variation in the success of firms. As discussed in the political context, this could be because CEOs do not matter, or it could be that firms have effective practices for hiring and incentivizing their CEOs such that, in equilibrium, most CEOs perform similarly.

When studying sports coaches, we focus on the high-stakes settings in the United States in which coaches are highly compensated—Major League Baseball (MLB), NBA, NHL, NFL, college football, and men’s college basketball. All data were provided by Sports Reference Inc. The outcome that is most readily observable and arguably most important is whether a game is won or last. Furthermore, we have data on the scores of each game, so we can also examine points scored, points allowed, and the point margin. In some sports, there are reasons to think that coaches might have more ability to affect points scored versus points allowed, or vice versa, so we separately examine both outcomes. For some sports, we have also collected additional data and conducted our test on other outcomes of specific interest for that sport.

We start with a dataset in which each unique game-team combination is an observation. To improve precision, we attempt to account for opponent quality and home-field advantage in the following way. For each outcome of interest, we calculate the average value for the opposing team across all games that season that were not played against the team in question. Then, we run a regression of the outcome of interest on these measures of opponent quality, year fixed effects, and an indicator for a home versus away game. We calculate the residuals from this regression, indicating the performance of each team in each game over above what would be expected given the year, home field advantage, and quality of their opponent. We then calculate the average residual across games for each team season, which becomes our outcome of interest for estimating coach effects. Aggregating up to the team season level causes little to no loss of information since the coach rarely changes mid-season. In the rare cases where this occurs, we assign the person who coached the most games that season to the entire season.

Results are shown in Table 4. We start with MLB managers, using data from 1871 to 2016. We find evidence that MLB managers matter for all four outcomes—runs scored, runs allowed, margin of victory, and victories—although they appear to matter more for runs allowed than for runs scored. For runs scored, the estimate is not statistically significant at conventional levels (*P* = 0.058), and the substantive magnitude of the estimated effect is smaller. However, for runs allowed, the *P* value is strongly statistically significant (*P* < 0.001) and substantively large, suggesting that managers explain 28% of the variation within teams and across seasons in runs allowed.

**Table 4:** Results for sports coaches. Avg, average permuted *r*^2^; Diff, the difference between the real *r*^2^ and the average permuted *r*^2^; Prop, the estimated proportion of the variation in an outcome explained by leader effects.

**Setting**	**Outcome**	***r*^2^**	**Avg**	**Diff**	***P***	**Prop**
MLB, 1871–2016	Runs scored	0.360	0.334	0.026	0.058	0.182
	Runs allowed	0.431	0.360	0.071	0.000	0.277
	Margin of victory	0.440	0.373	0.067	0.000	0.270
	Win	0.383	0.328	0.055	0.000	0.249
NFL, 1922–2016	Points scored	0.353	0.327	0.026	0.070	0.193
	Points allowed	0.362	0.314	0.048	0.002	0.253
	Margin of victory	0.392	0.347	0.045	0.002	0.245
	Win	0.350	0.318	0.032	0.015	0.211
CFB, 1900–2016	Points scored	0.360	0.307	0.052	0.000	0.257
	Points allowed	0.412	0.335	0.077	0.000	0.300
	Margin of victory	0.422	0.349	0.072	0.000	0.294
	Win	0.370	0.315	0.055	0.000	0.262
NBA, 1947–2017	Points scored	0.396	0.289	0.107	0.000	0.300
	Points allowed	0.387	0.311	0.077	0.000	0.283
	Margin of victory	0.422	0.314	0.108	0.000	0.300
	Win	0.415	0.315	0.100	0.000	0.300
CBB, 1938–2017	Points scored	0.374	0.269	0.104	0.000	0.300
	Points allowed	0.428	0.300	0.128	0.000	0.300
	Margin of victory	0.340	0.284	0.056	0.000	0.268
	Win	0.301	0.254	0.047	0.000	0.250
NHL, 1918–2017	Goals scored	0.404	0.358	0.046	0.016	0.232
	Goals allowed	0.458	0.375	0.083	0.000	0.298
	Margin of victory	0.464	0.387	0.077	0.000	0.288
	Win	0.432	0.372	0.059	0.004	0.259

One potential explanation for this discrepancy is that managing defense in baseball requires more strategic decisions than managing offense. For the most part, the job of the manager on offense is to put the best hitters in the lineup in the best order, and most managers would probably make similar decisions with the same team. However, on defense, the manager must efficiently use their pitchers without wearing out their arms. Some managers may be better than others at determining when a starter has thrown too many pitches to be effective, when to use a reliever, and which reliever to use in a particular situation.

One common notion is that a big part of an MLB manager’s job involves the allocation of scarce resources across games. Each team plays 162 games in the regular season, and each pitcher can only throw so many pitches per week. Therefore, some might argue that the most important job of the manager is not to increase runs scored or reduce runs allowed but instead to efficiently allocate runs across games. If the outcome of one game is a forgone conclusion, then a manager might as well save their best pitchers for the next game. To test whether some managers are better at this than others, we also use wasted runs as one of our outcomes of interest. We measure wasted runs as the margin of victory when a team wins and the number of runs scored when a team loses, and we might expect efficient coaches to reduce the number of wasted runs, allowing their teams to win more games with the same numbers of runs scored and allowed. We find little evidence that managers affect wasted runs. Our estimated effect of coaches on wasted runs in substantively small and statistically insignificant (detailed results available upon request). One potential explanation for this result is that it may not be easy to ex ante predict which runs will be wasted or not, and therefore, managers are unable to effectively decide when to save their pitchers for the next game.

Because we have so many years of data for the MLB, we have separately analyzed different eras, and we do not observe large changes over time in the importance of managers. However, we have separately analyzed the American and National leagues after 1973 to see whether the designated hitter rule appears to have changed the importance of managers. We find suggestive evidence that manager effects are greater in the National League, which did not adopt the designated hitter rule, and where a manager must make more strategic decisions regarding their batting and pitching lineup. However, we do not have enough data to draw statistically strong conclusions.

Table 4 also shows the results of our analyses for NFL coaches. We look at virtually the entire history of the NFL from 1922 to 2016, although the results are similar if we just focus on the modern era. As with baseball, we analyze points scored, points allowed, point margin, and victories. NFL coaches appear to affect all outcomes, although the estimate is statistically stronger and substantively larger for points allowed than for points scored.

We also have season-level data for some other outcomes of interest for the NFL during the modern era of 1970 to 2016. Specifically, we examine fumbles per game, penalties committed per game, opponents’ penalties per game, and the proportion of offensive plays on which a team passes (detailed results available upon request). We find that coaches matter a lot for fumbles and for the penalties a team commits. Coach effects explain about 30% of the within-team, between-year variation in these variables, with some coaches apparently doing a much better job preventing fumbles and penalties than others. Coaches appear to have little effect on penalties committed by opponents, perhaps revealing that there is not much a team can do to systematically induce penalties by their opponents. In addition, quite unexpectedly, coaches do not appear to meaningfully differ in their use of passing versus rushing. Coaches could simply force their teams to pass or run more often, but we do not find much evidence that coaches systematically differ from one another on this dimension. Perhaps, most coaches are following the same rules of thumb and/or are getting their teams close to the optimal share of passing versus rushing.

Table 4 also shows our results for college football coaches from 1900 to 2016. We include data from all Division 1-A teams after 1978, when Division 1 was subdivided, and all Division 1 teams before 1978. The estimated effects are larger for college football than for professional football. One potential explanation is that in addition to managing practices and games, college football coaches also play a crucial role in recruiting. Furthermore, because we have so much data for college football, the results are extremely statistically significant (*P* < 0.001) for all outcomes.

Next, we study coaches in the NBA and Division 1 men’s college basketball, respectively. In both cases, the estimated effects are substantively quite large. Coaches explain about 30% of the variation in points scored and allowed. One initially unexpected result is that in college basketball, coaches matter more for points scored and allowed than they do for the point margin. One potential explanation is that coaches differ from each other in their preferences for fast- versus slow-paced games, with the fast-paced coaches both scoring and allowing more points. To explicitly test this hypothesis, we have also tested whether coaches matter for the total points scored in the game (detailed results available upon request), and here, we detect a huge effect, confirming this hypothesis about different coaching styles.

Last, Table 4 shows results from NHL coaches from 1918 to 2017. As with the other sports, coaches matter and the results are statistically significant, and as with baseball and football, hockey coaches appear to matter more for goals allowed than for goals scored.

## DISCUSSION

Our method allows us to estimate the extent to which variation in performance is attributable to leaders as opposed to luck and other factors outside their control. We think that it is useful to know how much leaders matter and for what outcomes they matter most, but our method does not allow us to say which leaders are particularly effective or ineffective. Analysts and scholars will naturally want to know which leaders are most effective, and although our method is not suited for answering that question directly, our basic approach to inference can be useful for this question as well. Often, however, it will be difficult to confidently assess the quality of an individual leader. We illustrate this challenge by discussing the effectiveness of individual NFL coaches.

**Fig. 4:**
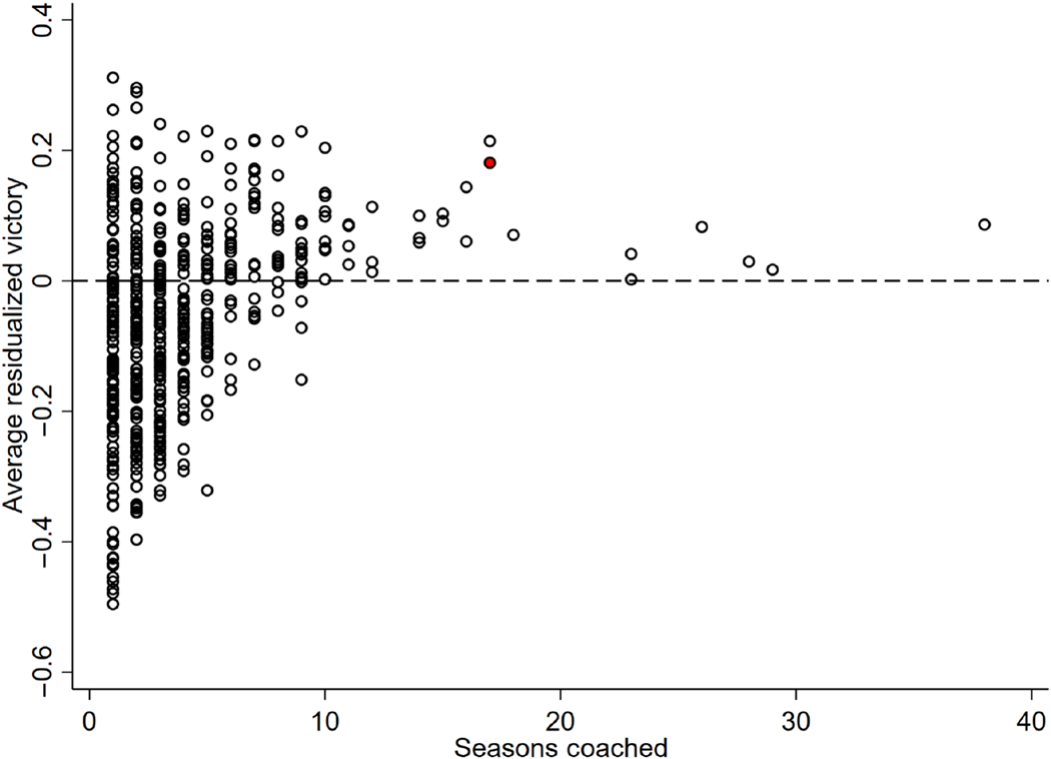
NFL coach performance across seasons coached. The figure plots the residualized probability of winning for every NFL coach across the number of seasons that they coached. Belichick is shown in red.

Assessing a leader’s ability is especially difficult when we only observe that leader serves for a short period of time. For example, if an NFL coach has a good record in their first few seasons, then this does not provide much information about their quality. Other factors were likely working in that coach’s favor, and we will expect mean reversion in subsequent years. To illustrate this point that the best leaders are not necessarily the ones with the best average levels of performance, Fig. 4 shows the average residual victory across seasons coached for every coach by team in the history of the NFL. The coaches with the best averages tend to be those that only coached one or two seasons, just like the coaches with the worst averages tend to have only coached one or two seasons. A few coaches have averages above 0.25, meaning that they were 25 percentage points more likely to win a game than an average coach, conditional on the quality of their opponent and home field advantage. This is a remarkable record. However, nobody who coached more than two seasons maintained such a high record. In addition, furthermore, even if coaches did not matter, we would probably expect a few coaches to have records like this merely by chance.

To identify the coaches that are genuinely likely to be much better than average, we would want to look at those who coached more seasons, and we would want to look at those coaches on the upper frontier who have impressive records given their number of seasons in the league. Some analysts believe that Belichick is one of the greatest NFL coaches of all time, and his remarkable tenure with the New England Patriots is filled in and colored red in Fig. 4. Through the 2016 season for which we have data available, Belichick had served 17 seasons with the Patriots, and his average residual victory was 0.18. Other coaches have higher averages, but an average that high after coaching so many seasons is extremely unusual. Only Paul Brown’s 17-year tenure with the Cleveland Browns starting in 1946 exceeds Belichick’s run.

How can we test whether an outlier like Belichick is genuinely great or whether he could have achieved his success by luck? Similar in spirit to our previously discussed Monte Carlo simulations, we could simulate outcomes in a world in which all coaches are equally effective but there is random noise, serial correlation, and endogenous turnover, and we could see how often someone who looks as good as Belichick arises. The right question is not the odds that a randomly selected coach will look as good as Belichick. Belichick might just be the luckiest coach, and we are focusing on him because of his impressive record. Instead, the right question is about the odds that any coach could arise with a record as good as Belichick’s even in a world where coaches do not matter.

Using endogenous turnover simulations from Table 1 and using the parameters estimated for the NFL, we can compute the average performance in their first 17 seasons for all simulated coaches that served that long, and we can record the best such average across all coaches. We can then compare this to Belichick’s actual performance, and we can see how likely such a record is to arise by chance. The answer is very unlikely.

**Fig. 5:**
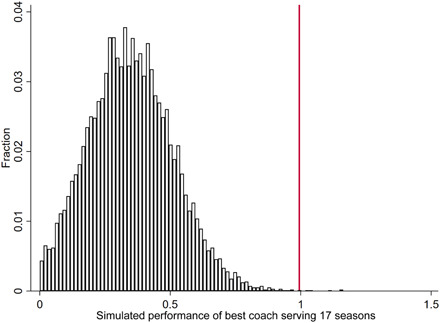
Hypothesis test of Belichick’s effectiveness. The figure shows the simulated distribution of the career performance of the best coach who served for 17 seasons in a hypothetical world with no coach effects. Belichick’s actual performance after 17 seasons is indicated by the red line.

The results of this exercise are shown in Fig. 5. If we standardize the season-level performance measure, then we see that Belichick’s season-level average is one SD above the mean. When we simulate 10,000 hypothetical NFLs with no coach effects and with serial correlation and endogenous retention comparable to what we observe in the real data, there are only five cases in which a coach served 17 seasons and had such an impressive record. In that sense, we can strongly reject the null hypothesis that Belichick is no better than an average coach (*P* = 0.0005).

To illustrate the difficulty of assessing a coach’s quality early in their careers, we have conducted the same kinds of simulations but only using data from an early point in a coach’s career. The best average residual victory for any coach in their first season was 0.412, achieved by Adam Walsh who coached the Rams in 1945. In our simulations with no coach effects, at least one coach has a first season as good as this one 62% of the time. The null result for Walsh may be informative since he had a lackluster season in 1946 and then never coached at the professional level again.

Similarly, the best first two seasons for any coach were achieved by George Seifert with the 49ers in 1989 and 1990. In our simulations with no coach effects, at least one coach exceeded Seifert in their first two seasons 57% of the time. This might be a false negative since Seifert won two Super Bowls and only missed the playoffs once in his eight seasons with the 49ers. However, as coach of the Panthers, Seifert had two lackluster seasons and one atrocious season before being fired, so we may have been right not to draw overly strong conclusions from two great seasons.

Using a conventional threshold of 0.05 for statistical significance, we cannot reject the null hypothesis that any NFL coach is better than average if we only use data from their first four seasons. We have to wait until they have served five seasons before we are able to find statistically significant evidence for any coach. The highest average by any coach in their first five seasons was achieved by the aforementioned Paul Brown, and for him, we obtain a *P* value of 0.008 at that point in his career. Sure enough, Brown went on to have a long, successful career with Cleveland and Cincinnati, maintaining a better-than-average record throughout.

Future analysts could adapt this procedure to their setting of interest and apply this logic to determine whether we should be confident that a particular leader is truly effective. The method cannot explicitly say which leaders are best, but it can assess which leaders’ records are more or less likely to have arisen by chance, which is informative for forecasting future success. However, for a democratically elected leader who might have only served one 4-year term, it will typically be difficult to know if their success (or lack thereof) was attributable to talent versus luck.

We have proposed a new approach for estimating leader effects—RIFLE. The primary innovation relies on random permutations for inference. Relative to methods previously used in the literature, ours makes use of more variation in the data and relies on fewer and weaker assumptions. In Monte Carlo simulations, RIFLE performs well even in relatively small samples. In addition, our method does not require knowledge of the particular circumstances surrounding leader transitions or the cause of leader deaths in office, making our approach easier to generalize to subnational or other less prominent offices where leader biographies are unlikely to be available.

Consistent with previous evidence, we show that world leaders matter for economic growth and they matter more autocracies than democracies. Inconsistent with previous evidence, we find little evidence that CEOs matter. We also apply our method to settings where leader effects had not previously been estimated. We find no evidence that U.S. governors and mayors affect income and employment in their jurisdictions. We do find that governors, but not mayors, influence public finance in the forms of expenditure and federal aid. In addition, we also find some evidence that governors, but not mayors, affect crime in their jurisdictions. We also find that strong evidence that sports coaches matter, more so than political or business leaders. We hope the application of our method to different contexts will further improve our general understanding of where, when, and why leaders matter.

## Supplementary Material

http://advances.sciencemag.org/cgi/content/full/7/4/eabe3404/DC1

Adobe PDF - abe3404_SM.pdf

Leadership or luck? Randomization inference for leader effects in politics, busines, and sports

## SUPPLEMENTARY MATERIALS

Supplementary material for this article is available at http://advances.sciencemag.org/cgi/content/full/7/4/eabe3404/DC1
