# Supplementary material for: Leadership or luck? Randomization inference for leader effects in politics, business, and sports
Source: Sci Adv. 2021 Jan 20;7(4):eabe3404. doi: 10.1126/sciadv.abe3404 (PMC7817108; doi:10.1126/sciadv.abe3404)
Supplement: http://advances.sciencemag.org/cgi/content/full/7/4/eabe3404/DC1 [file supp_7_4_eabe3404__index.html]

Science Advances | Science AdvancesAAASSearchScience AdvancesMenu

## Supplementary Materials

# Leadership or luck? Randomization inference for leader effects in politics, business, and sports

Christopher R. Berry and Anthony Fowler

Download Supplement

**This PDF file includes:**

- Text S1
- Fig. S1

**Files in this Data Supplement:**

- Adobe PDF - abe3404\_SM.pdf
