# Supplementary material for: Leadership or luck? Randomization inference for leader effects in politics, business, and sports
Source: Sci Adv. 2021 Jan 20;7(4):eabe3404. doi: 10.1126/sciadv.abe3404 (PMC7817108; doi:10.1126/sciadv.abe3404)
Supplement: http://advances.sciencemag.org/cgi/content/full/7/4/eabe3404/DC1 [file supp_7_4_eabe3404__1.pdf]

Science Advances
AAAS

advances.sciencemag.org/cgi/content/full/7/4/eabe3404/DC1

# Supplementary Materials for

## Leadership or luck? Randomization inference for leader effects in politics, business, and sports

Christopher R. Berry and Anthony Fowler*

*Corresponding author. Email: anthony.fowler@uchicago.edu

Published 20 January 2021, *Sci. Adv.* **7**, eabe3404 (2021)
DOI: 10.1126/sciadv.abe3404

**This PDF file includes:**

Text S1
Fig. S1

**Text S1**

Leadership or Luck:

Randomization Inference for Leader Effects in Politics, Business, and Sports

## Transition Costs

To explore the implications of transition costs for our test, we conducted an additional battery of Monte Carlo simulations. For simplicity, everything is identical to the analyses in Figure 1 with 20 units and 20 periods except there is no serial correlation and there is a transition cost in the first period each leader takes office. Specifically, the outcome in each period is drawn from a normal distribution with a mean of 0 and standard deviation of 1, and a constant amount is subtracted in transition periods. This means that in non-transition years, the mean and standard deviation are 0 and 1, respectively, while in transition years the mean and standard deviation are −X and 1, respectively, where X corresponds to the magnitude of the transition cost. In Figure S1, we show results for transition costs of .1, .25, .5, 1, and 2.

For each transition cost, we simulate 1,000 data sets and implement RIFLE along with a test in the spirit of Jones and Olken (2005). When implementing the latter test, we compute the absolute change in growth for each period, i.e., $|Y_t - Y_{t-1}|$, and we test whether this absolute change is greater in transition versus non-transition years using a t-test.

Figure S1 shows the distribution of p-values resulting from both tests across different transition costs. As expected, the Jones and Olken test (top row of Figure S1) produces p-values that are skewed right, meaning that this test over-rejects the null hypothesis. Furthermore, the transition cost need not be too large to generate a significant bias. On the other hand, RIFLE performs much better in the presence of transition costs. Unless the transition cost is larger than

**Figure S1. Effect of Transition Costs for Jones and Olken vs. RIFLE**

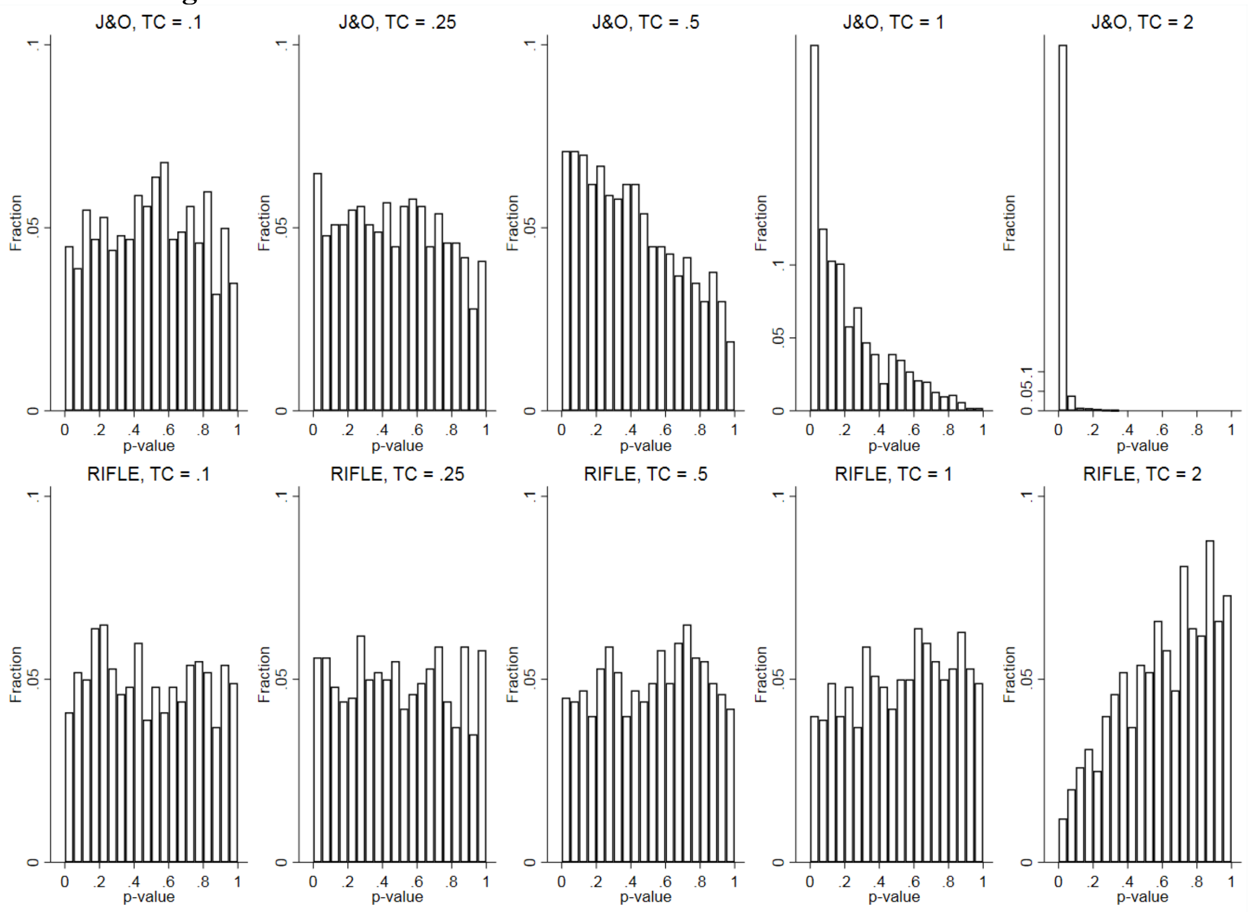


*Each histogram shows the distribution of p-values resulting from 1,000 simulated data sets with transition costs (TC) of varying magnitudes. The top row presents results from a test in the spirit of Jones and Olken (2005), and the bottom row presents tests using RIFLE. See the text for more details.*

the standard deviation of the outcome in non-transition years, the bias introduced by transition costs is negligible. Furthermore, when a bias is detectable, it goes in the opposite direction. Specifically, the distribution of p-values is skewed to the left, meaning that RIFLE under-rejects the null. The intuition for this result is provided in the main text.

The test of Jones and Olken overstates leader effects in the presence of transition costs, while RIFLE performs much better. When there is a meaningful bias for RIFLE, which only occurs for very large transition costs, it leads us to understate leader effects. Overall, the implications of

transition costs are minimal for RIFLE, and if anything, they lead RIFLE to be a conservative test of leader effects.

**Theoretical Model of Endogenous Turnover**

As discussed in the main text, one concern for our test is that the outcome of interest influences the tenures of leaders. Suppose, for example, that governors do not matter for a particular outcome, but voters believe that they do, and therefore, the values of that outcome variable influence the chances that the governor will stay in office. This kind endogenous turnover could potentially bias the results of RIFLE.

To understand and illustrate the bias that arises from endogenous turnover, consider the following theoretical model. Suppose there is a binary outcome of interest, all leaders are the same (i.e., the outcome is unrelated to the identity of the leader), there is a two-term limit, and turnover for first-termers depends entirely on the outcome in the first term.

Recall that $r^2 \equiv 1 - \frac{RSS}{TSS}$, where RSS is the residual sum of squares and TSS is the total sum of squares. With RIFLE, the TSS is identical for both the real data and the permuted data sets where the ordering of leaders is randomly shuffled. Therefore, to think about how RIFLE will perform, we can focus on the RSS. Under the null, we'd like the RSS to be identical, in expectation, for the real data and the permuted data. If the expected RSS is greater in the real data, that means the r-squared will be smaller, and we will under-reject the null. If the expected RSS is smaller in the real data, the r-squared will be larger, and we will over-reject the null. Since the sample size is the same for both the real and permuted data sets, we can also think about the average squared residual, and by comparing the expected average squared residual in the real and the permuted data, we can assess whether RIFLE will over- or under-reject the null.

In this theoretical model, the data set of leaders and outcomes will include only three different kinds of leaders. Anyone who has a bad year in their first term will be removed from office, so there will be one-termers who had a bad outcome—let's refer to this type of leader as 0. To serve two terms, the outcome must have been good in the first term, but the outcome could have been either good or bad in the second term, so in addition to 0's there are also 1-0's and 1-1's.

Because leaders don't matter, the probability of a good outcome does not depend upon who is in office. Let's call the probability of a good term $p$, where $0 < p < 1$. In expectation, the proportion of 0's among all leaders is $1 - p$, the proportion of 1-0's is $p(1 - p)$, and the proportion of 1-1's is $p^2$. As a share of all observations (i.e., periods) in the data set, 0's comprise $\frac{1-p}{1+p}$ of them, 1-0's comprise $\frac{2p-2p^2}{1+p}$, and 1-1's comprise $\frac{2p^2}{1+p}$.

Let's calculate the expected average squared residual in the real data. The squared residual for the 0's and the 1-1's is 0. In both cases, their leader fixed effect perfectly fits every data point. For the 0-1's, the predicted values will be $\frac{1}{2}$, the residuals will be $\frac{1}{2}$, so the squared residuals will be $\frac{1}{4}$. Since 1-0's comprise $\frac{2p-2p^2}{1+p}$ of all observations in our data set, the expected average squared residual in the real data set is $(\frac{1}{4})(\frac{2p-2p^2}{1+p}) = (\frac{1}{2})(\frac{p-p^2}{1+p})$.

In our random permutations, instead of three kinds of leaders, there will now be six: 0, 1, 0-0, 0-1, 1-0, and 1-1. Four of these types have no variation in their outcome so they make no contribution to the RSS, whereas the 0-1's and the 1-0's will again have residuals equal to $\frac{1}{4}$. Those two types will comprise $2p^2(1 - p)$ of the leaders and $\frac{4p^2(1-p)}{1+p}$ of the terms. Therefore, the expected average squared residual in the permutated data set is $\left(\frac{1}{4}\right)\left(\frac{4p^2(1-p)}{1+p}\right) = p(\frac{p-p^2}{1+p})$.

Comparing the expected average squared residuals in the real and permuted data, we see that they equal each other if and only if $p = \frac{1}{2}$. If $p > \frac{1}{2}$, the squared residuals will be greater in the permuted data, meaning the r-squared is lower, and RIFLE will over-reject the null. Alternatively, if $p < \frac{1}{2}$, the squared residuals will smaller in the permuted data, meaning the r-squared will be greater, and RIFLE will under-reject the null. In other words, endogenous turnover can produce a bias, and that bias can go in either direction.

To gain some intuition for the bias in the model, consider an extreme case where $p$ is very close to zero but still positive. Remember that the r-squared of the regression of the outcome on leader fixed effects is determined entirely by the proportion of two-term leaders that have one good term and one bad term. In the rare case when there is a good outcome and a leader is retained, they will almost certainly be a 1-0. 1-1's will be exceedingly rare relative to 1-0's. This means that almost all of the two-termers in the real data will be 1-0's, contributing positively to the RSS. When we permute the leader tenures, most of those two-term leaders will happen fall on two bad terms, and they'll become 0-0's, where they will not add to the RSS. The r-squared will be very high in both cases, but it will be almost exactly 1 in the permuted data, and it will be slightly lower in the real data, meaning that RIFLE will under-reject the null.

Similarly, consider an extreme case where $p$ if very close to but still less than 1. Almost all leaders are 1-1's, and there are roughly equal shares of 0's and 1-0's. When there is a rare bad outcome, half of those belong to one-termers, in which case they contribute nothing to the RSS. However, most of the leaders are two-termers, which means that in the random permutations, most of the bad outcomes get assigned to two-termers, creating 1-0's or 0-1's and increasing the RSS. Again the r-squared is very high in both cases, but it's higher in the real data than the permuted

data, meaning that we over-reject the null. Fortunately, the Monte Carlo simulation results in Table 1 show that the extent of this bias is likely small in more realistic scenarios.
